# Human Neutrophil Lipocalin in Activated Whole Blood Is a Specific and Rapid Diagnostic Biomarker of Bacterial Infections in the Respiratory Tract

**DOI:** 10.1128/CVI.00064-17

**Published:** 2017-07-05

**Authors:** Per Venge, Ann-Katrin Eriksson, Lena Douhan-Håkansson, Karlis Pauksen

**Affiliations:** a Departments of Medical Sciences and Clinical Chemistry, Uppsala University, Uppsala, Sweden; b Department of Infectious Disease, Uppsala University, Uppsala, Sweden; c Diagnostics Development, Uppsala University, Uppsala, Sweden; IIS/LAD/NIAID/NIH

**Keywords:** antibiotic resistance, biomarker, lipocalin, point of care, respiratory infection

## Abstract

The distinction between bacterial and viral causes of infections of the respiratory tract is a major but important clinical challenge. We investigated the diagnostic performance of human neutrophil lipocalin (HNL) in respiratory tract infections compared to those of C-reactive protein (CRP) and procalcitonin (PCT). Patients were recruited from the emergency department and from a primary care unit (*n* = 162). The clinical diagnosis with regard to bacterial or viral cause of infection was complemented with objective microbiological/serological testing. HNL was measured in whole blood after preactivation with the neutrophil activator formyl-methionine-leucine-phenylalanine (fMLP) (B-HNL), and CRP and PCT were measured in plasma. Head-to-head comparisons of the three biomarkers showed that B-HNL was a superior diagnostic means to distinguish between causes of infections, with areas under the concentration-time curve (AUCs) of receiver operating characteristic (ROC) analysis for HNL of 0.91 (95% confidence interval [CI], 0.83 to 0.96) and 0.92 (95% CI, 0.82 to 0.97) for all respiratory infections and for upper respiratory infections, respectively, compared to 0.72 (95% CI, 0.63 to 0.80) and 0.68 (95% CI, 0.56 to 0.79) for CRP, respectively (*P* = 0.001). In relation to major clinical symptoms of respiratory tract infections (cough, sore throat, stuffy nose, and signs of sinusitis), AUCs varied between 0.88 and 0.93 in those patients with likely etiology (i.e., etiology is likely determined) of infection, compared to 0.63 and 0.71 for CRP, respectively, and nonsignificant AUCs for PCT. The diagnostic performance of B-HNL is superior to that of plasma CRP (P-CRP) and plasma PCT (P-PCT) in respiratory tract infections, and the activity specifically reflects bacterial challenge in the body. The rapid and accurate analysis of HNL by point-of-care technologies should be a major advancement in the diagnosis and management of respiratory infections with respect to antibiotic treatment.

## INTRODUCTION

The distinction between bacterial and viral causes of infections of the respiratory tract is a major clinical challenge, which often leads to the unnecessary prescription of antibiotics. As aids in diagnosis, white blood cell counts and plasma levels of C-reactive protein (CRP) have been widely used for many years. However, although rapid, none of these tests have met the requirement of being sufficiently accurate, which has prompted the need in many cases to perform microbiological tests. In our previous reports, we showed that human neutrophil lipocalin (HNL) might fulfill this unmet clinical need, since the diagnostic performances of both serum HNL and HNL released in whole blood (B-HNL) after activation with the tripeptide formyl-methionine-leucine-phenylalanine (fMLP) were superior to the performances of both contemporary biomarkers and novel biomarkers, such as procalcitonin and CD64 expression in neutrophils (1–4). In the previous Bio-X study, the results of CRP were available in the clinical judgment of the patient, which means that the diagnostic performance of CRP might be positively biased. We therefore reexamined the performance of CRP and chose to illustrate this in the subcohort of patients with respiratory infections.

In the clinical study of Bio-X, we preincubated whole blood for 20 min before assaying the release of HNL (3). A second objective of this report was to study the very early kinetics of fMLP release from neutrophils in whole blood and to examine the feasibility of producing an assay of HNL with a response time of a few minutes, therefore being suitable for rapid point-of-care application.

## RESULTS

**FIG 1:**
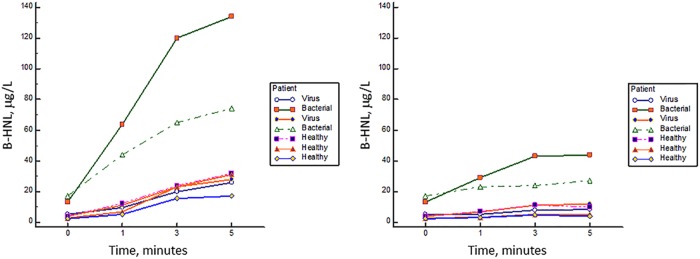
The kinetics of HNL release in whole blood after fMLP activation (left) and without activation (right). Blood samples from patients with bacterial or viral infections and healthy controls were investigated.

In Fig. 1, the release of HNL after activation of whole blood with fMLP is shown. It is seen that HNL release is highly increased already at 1 min of incubation of fMLP in blood from patients with acute bacterial infections, in contrast to the findings in whole blood from healthy subjects and from patients infected by a virus. After 5 min of incubation, a slight increase was seen in the blood from healthy noninfected subjects and in those with viral infections, whereas the increase in the blood from those with bacterial infections was substantially higher. In the right panel of Fig. 1, we show the release of HNL in the absence of activator. In one of the patients with an acute bacterial infection, some release occurred in whole blood without activator.

As indicated in Materials and Methods, the diagnosis of a group of our patients was judged to be have bacterial or viral cause, based on clinical findings. Table 1 shows the concentrations of plasma CRP (P-CRP), B-HNL, and plasma PCT (P-PCT) in patients with respiratory infections and in whom a clinical diagnosis as to bacterial or viral infection was judged from clinical findings; it also shows the results from those patients in whom the diagnosis was likely based on objective (i.e., microbiological) findings. The differences between the two groups were highly significant for all comparisons (*P* < 0.001), except for the comparison of the PCT concentrations in bacterial and viral infections with a likely diagnosis based on objective findings. The diagnostic performances of CRP, HNL, and PCT were judged by receiver operating characteristic (ROC) curve analysis, and the results from CRP and HNL are shown in Table 2. In the group in which diagnosis was based on clinical findings only, the areas under the concentration-time curve (AUCs) of CRP and HNL were similar, whereas in the group in which the diagnosis was based on clinical findings and further objective testing, the AUC of CRP dropped, whereas the AUC of HNL increased to 0.91 and was significantly different from the AUC of CRP (*P* = 0.001). For HNL, the positive likelihood ratio (LR+) increased from 4.4 to 9.9, and the negative likelihood ratio (LR−) decreased from 0.29 to 0.19. For CRP, the LR+ was almost unchanged, but the LR− increased from 0.42 to 0.54. For PCT, the ROC curve analysis showed an AUC of 0.67 (95% confidence interval [CI], 0.59 to 0.74), LR+ of 3.9, and LR− of 0.73 in patients with a clinical diagnosis of their respiratory infections, whereas the AUC in patients with likely etiology was 0.57 (95% CI, 0.47 to 0.67), the LR+ was 2.2, and the LR− was 0.76.

**TABLE 1:** Concentrations of P-CRP, B-HNL, and P-PCT in patients with respiratory infections and clinical diagnosis as to bacterial or viral cause^*a*^

Biomarker	Clinical diagnosis				Clinical diagnosis and likely microbiological etiology			
	Bacterial		Viral		Bacterial		Viral	
	*n*	Concn (range)	*n*	Concn (range)	*n*	Concn (range)	*n*	Concn (range)
P-CRP	97	115 (43–203)	70	23 (9–45)	75	94 (41–197)	31	44 (21–68)
B-HNL	77	309 (206–437)	46	152 (114–172)	57	303 (211–494)	25	137 (108–161)
P-PCT	98	0.132 (0.061–0.502)	70	0.076 (0.045–0.142)	76	0.133 (0.105–0.255)	30	0.124 (0.067–0.216)

a Concentrations for P-CRP are given in milligrams per liter, and concentrations for B-HNL and P-PCT are in micrograms per liter. The differences in concentrations between bacterial and viral infections were highly significant for all comparisons (*P* < 0.001), except for the comparison of PCT concentrations in bacterial and viral infections with likely etiology.

**TABLE 2:** ROC analysis of the distinction between bacterial or viral causes of respiratory infections

Characteristic	Clinical diagnosis	Clinical diagnosis and likely microbiological etiology
P-CRP		
*n*	166	104
AUC (95% CI)	0.81 (0.74–0.87)	0.72 (0.63–0.80)
Likelihood ratios^*a*^		
LR+	6.2	5.0
LR−	0.42	0.54
Optimal concn (mg/liter)	72	95
B-HNL		
*n*	123	86
AUC (95% CI)	0.83 (0.75–0.89)	0.91 (0.83–0.96)
Likelihood ratios^*a*^		
LR+	4.4	9.9
LR−	0.29	0.19
Optimal concn (μg/liter)	202	189
*P* value (comparison of AUCs)	NS^*b*^	0.001

a Likelihood ratios based on the Youden index.

b NS, nonsignificant.

**FIG 2:**
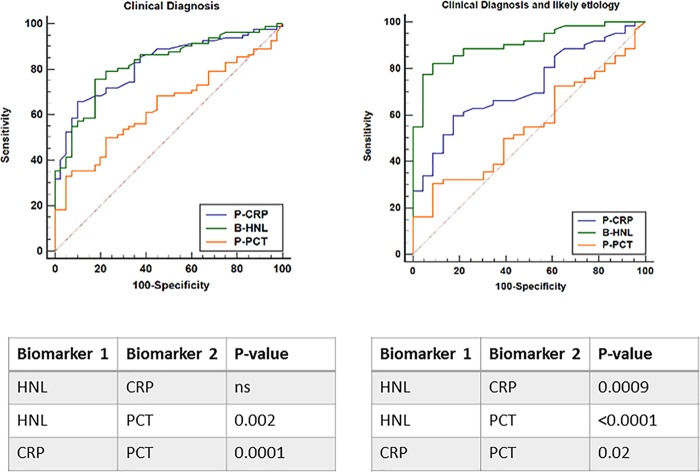
The distinction between bacterial and viral causes of respiratory tract infections. Left panel shows the ROC curves based on clinical diagnosis of causes of the infections, and right panel the ROC curves based on clinical diagnosis and likely microbiological etiology of the infections. Below the graphs, the statistical differences between the biomarkers are shown and given as *P* values. ns, nonsignificant.

In Fig. 2, the comparisons among the three biomarkers are shown head to head. The findings were similar to the results given in Table 2. Thus, in patients with likely etiology, the AUC of HNL separated clearly from the AUCs of CRP and PCT (*P* = 0.0009 and <0.0001, respectively).

**FIG 3:**
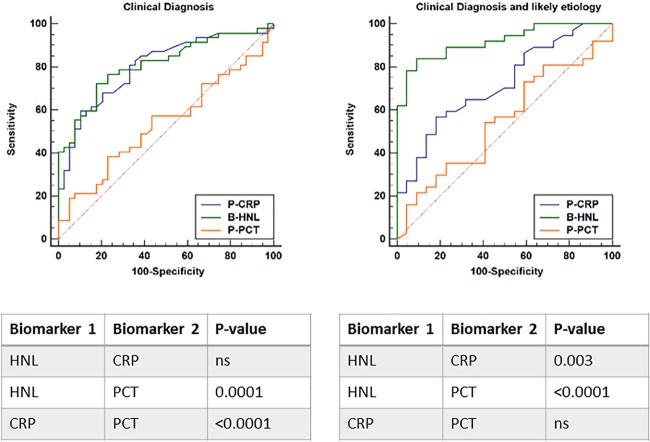
The distinction between bacterial and viral causes of upper respiratory tract infections. Left panel shows the ROC curves based on clinical diagnosis of causes of the infections, and right panel the ROC curves based on clinical diagnosis and likely microbiological etiology of the upper respiratory infections. Below the graphs, the statistical differences between the biomarkers are shown and given as *P* values.

In Table 3, we examined the results in patients with upper respiratory infections and found similar diagnostic performances of CRP, HNL, and PCT, as described above; i.e., with the inclusion of all patients with clinical diagnosis, the AUCs of CRP and HNL were similar but different from that of PCT (not shown). However, with the inclusion of the patients with likely etiology of their upper respiratory infection, only the diagnostic performance of HNL was superior, with an LR+ of 9.6 and LR− of 0.18, compared to 4.1 and 0.62 for CRP, respectively. For PCT, the clinical performance in terms of AUC was nonsignificant. In Fig. 3, we show the head-to-head comparison among the three biomarkers, with results similar to those shown in Table 3, i.e., a highly significant difference in AUCs between HNL and CRP or PCT in patients with likely etiologies of their upper respiratory infections (*P* = 0.006 and <0.0001, respectively).

**TABLE 3:** ROC analysis of the distinction between bacterial or viral cause of upper respiratory infections

Characteristic	Clinical diagnosis	Clinical diagnosis and likely microbiological etiology
P-CRP		
*n*	115	69
AUC (95% CI)	0.78 (0.70–0.86)	0.68 (0.56–0.79)
Likelihood ratios^*a*^		
LR+	6.8	4.1
LR−	0.48	0.62
Optimal concn (mg/liter)	71	95
B-HNL		
*n*	87	60
AUC (95% CI)	0.81 (0.72–0.89)	0.92 (0.82–0.97)
Likelihood ratios^*a*^		
LR+	4.1	9.6
LR−	0.34	0.18
Optimal concn (μg/liter)	202	189
*P* value (comparison of AUCs)	NS^*b*^	0.001

a Likelihood ratios based on the Youden index.

b NS, nonsignificant.

**FIG 4:**
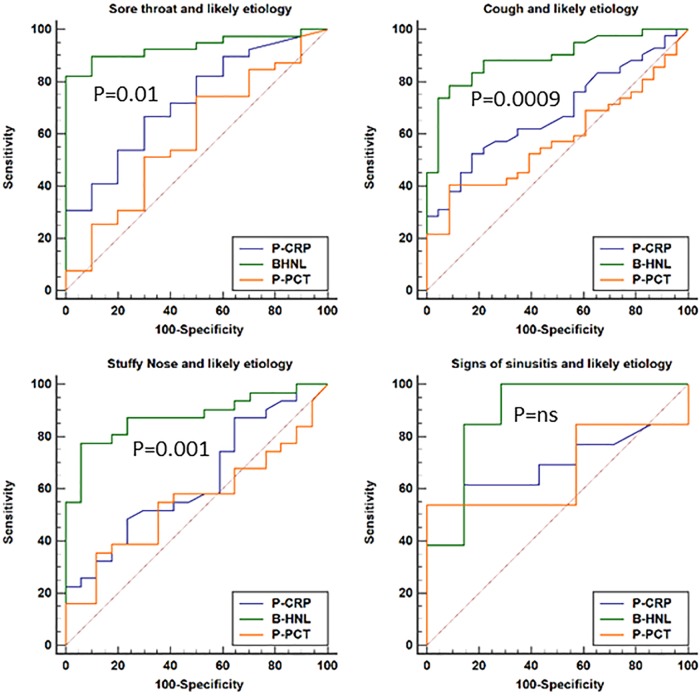
The distinction between bacterial and viral causes of infections in relation to symptoms of the respiratory tract. The results from patients with clinical diagnosis and likely microbiological etiology of their infections are shown. *P* values given on the figures are statistical differences between the ROC curves of B-HNL and P-CRP.

In Table 4, the diagnostic distinctions between bacterial and viral causes of the infections in relation to major clinical symptoms of cough, sore throat, stuffy nose, and signs of sinusitis are shown. CRP and HNL showed similar clinical performances when all patients were included (not shown), whereas PCT had lower AUCs with regard to all clinical symptoms than did CRP and B-HNL (*P* = 0.06 to 0.01). Examining patients with a likely etiology for their symptoms showed that the clinical performance of B-HNL increased to AUCs of 0.88 to 0.93, in contrast to CRP and PCT (Table 4). The AUCs of PCT were nonsignificant in relation to all four clinical symptoms. The head-to-head comparisons of the three biomarkers in relation to major clinical symptoms of the upper respiratory tract are shown in Fig. 4 and show results similar to those given in Table 4.

**TABLE 4:** ROC analysis of the distinction between bacterial or viral causes of infections with likely microbiological etiology in relation to major clinical symptoms

Characteristic	Cough	Sore throat	Stuffy nose	Sinusitis signs
P-CRP				
*n*	81	59	57	23
AUC (95% CI)	0.69 (0.57–0.79)	0.71 (0.58–0.82)	0.66 (0.52–0.78)	0.63 (0.41–0.82)
Likelihood ratios^*a*^				
LR+	4.4	3.2	2.9	4.3
LR−	0.59	0.59	0.67	0.53
Optimal concn (mg/liter)	95	95	90	60
B-HNL				
*n*	66	50	48	21
AUC (95% CI)	0.89 (0.79–0.96)	0.93 (0.82–0.98)	0.88 (0.75–0.95)	0.89 (0.68–0.99)
Likelihood ratios^*a*^				
LR+	9.4	>10.0	13.2	6.8
LR−	0.23	0.18	0.24	0.18
Optimal concn (μg/liter)	189	178	200	166
*P* value (comparison of AUCs)	0.0009	0.01	0.001	NS (0.06)^*b*^

a Likelihood ratios based on the Youden index.

b NS, nonsignificant.

## DISCUSSION

In our previous reports, we showed that HNL in serum (1, 4, 5) or as measured after activation of whole blood (2, 6) was superior to any other known biomarker, such as procalcitonin in plasma or CD64 expression in neutrophils, in the distinction between bacterial and viral causes of acute infections. Such data make HNL a very interesting candidate for the future management of acute infections in order to avoid the abuse of antibiotics (7, 8). However, the most widely used biomarker in the management of acute infections is currently CRP, and CRP has indeed been a valuable tool in the management of patients with acute infections (9). However, it is also well known that CRP will react to almost any process in the body that involves inflammation, which makes CRP quite unspecific. In our previous Bio-X study, CRP was known to the adjudicator and used to classify patients as having a bacterial or viral cause of infection. Thus, a bias toward CRP as a diagnostic principle was obvious. It is also seen in this report that the clinical performance of HNL seems no better than that of CRP in the diagnostic distinction between bacterial and viral infections. It was therefore of considerable interest that the diagnostic performance of HNL became superior to that of CRP when these biomarkers were evaluated in the cohort in which the likely cause of the infection was verified by objective microbiological testing. Thus, the diagnostic performance of CRP dropped, whereas the performance of HNL increased considerably, which is a strong support to the specificity of HNL as a reflection of bacterial infections. The diagnostic patterns between CRP and HNL were essentially identical whether all patients with respiratory infections were included or only patients with upper respiratory infections were examined. We also confirmed that PCT did not add much to the diagnostic distinction between bacterial and viral causes of acute respiratory infections (10–12), although numerous publications have advocated PCT as a tool to manage the antibiotic treatment of such patients (13–15). It is possible that the severity of our patients' infections is less than in many of those studies, although our previous results on sepsis also showed the diagnostic superiority of HNL over PCT (16). The potential clinical usefulness of HNL in the diagnosis and management of acute infections was also exemplified in this study by relating the concentrations to the major clinical symptoms of the patients. For example, in patients with a sore throat, the likelihood of a viral cause of disease was very low if HNL concentrations were increased. This was in spite of the fact that patients with streptococcal tonsillitis were excluded from this group. Similar results were seen with the other major symptoms, such as cough, stuffy nose, and signs of sinusitis. The superior diagnostic performance of B-HNL in upper respiratory infections is of considerable interest, since these are the most common symptoms and complaints of patients seeking medical care, at least in the primary care setting (17).

In our previous study from China, we saw a similar pattern, with HNL in serum being superior to CRP (1). In that study, CRP was not biased but was analyzed retrospectively. However, even if the clinical performance of HNL based on serum is very powerful, the drawback of serum measurements is obvious, since it takes several hours for the preanalytical step to prepare serum and additional time to measure HNL. Thus, for an emergency situation, a rapid point-of-care application is most attractive. Therefore, we extended previous experiments (6) on the kinetics of the release of HNL from blood neutrophils to very short incubations with the neutrophil activator fMLP. These experiments clearly support the potential to construct point-of-care assays with very short response times, i.e., in the range of 5 to 10 min, which should be the goal to satisfy the clinical requirement of fast and accurate decisions as to whether to treat infected patients with antibiotics or not.

HNL is a fairly complicated molecule, with several origins and several names, i.e., neutrophil gelatinase-associated lipocalin (NGAL) and lipocalin 2 (18). In blood, the major origin of HNL is blood neutrophils, in which HNL exists in a preformed state (19, 20). The production of HNL may, however, be induced in epithelial cells, such as in the kidney tubular cells in patients with acute kidney injury (AKI) (21, 22). If measured in serum or plasma, such production of HNL could potentially affect the diagnostic performance of HNL. HNL originating from neutrophils is, to a large extent, released in the dimeric form, whereas HNL released from epithelial cells is in the monomeric form. This means that assays should be able to distinguish these forms for optimal diagnostic performance. As shown recently, configuration of the assay by certain antibody pairs enables such distinction when measured in serum or plasma (1). In the present report, we have circumvented these potential confounders by measuring directly what is released from the circulating neutrophils.

One limitation of our study is the accuracy of the diagnosis, i.e., in the distinction between bacterial and viral causes of respiratory infections. Such a distinction is notoriously difficult (9) but very important if one wishes to investigate any biomarker for these purposes. In this report, we made an effort to make an accurate diagnosis by microbiologic and serologic testing of various body fluids, but with even more extensive testing, we might have reached higher diagnostic accuracies of HNL of ≫0.9. However, what seems very important in our study is that the diagnostic performance of HNL in the head-to-head comparison with CRP and PCT showed such a dramatic improvement when objective testing was included in the determination of diagnosis. As mentioned above, we take this as a very strong support to our notion that the measurement of HNL by specific assay formats is a reflection of the bacterial challenge of the body. Another limitation of this study is the diagnosis of the lower respiratory tract infections. Thus, we cannot exclude that some of the patients in whom X-ray examinations of the lungs were not indicated for clinical reasons, and therefore were not performed, actually had infections of the lower respiratory tract. This limitation, however, did not affect the overall conclusion of HNL being a superior diagnostic biomarker in respiratory infections.

We conclude that the head-to-head comparison among B-HNL, CRP, and PCT in the distinction between bacterial or viral cause of acute respiratory infections has demonstrated the superiority of B-HNL in this respect. Thus, the strength of our study is the fact that the biomarkers were judged against each other and that the diagnostic limitations affected the biomarkers equally.

## MATERIALS AND METHODS

In total, 581 patients with signs and symptoms of acute infections were recruited, as described previously (2, 6). One hundred sixty-two patients presented with symptoms of a respiratory tract infection were judged on clinical grounds to have either a bacterial (*n* = 98) or viral (*n* = 64) cause of their infection, and they were given a clinical diagnosis. The 98 patients with clinical diagnosis of bacterial infection comprised 55 women (median age, 37 years; range, 18 to 84 years) and 43 men (median age, 48 years; range, 20 to 90 years). The 64 patients with clinical diagnosis of viral infections comprised 36 women (median age, 40 years; range, 19 to 79 years) and 28 men (median age, 49 years; range, 30 to 92 years), with a significant difference in age (*P* = 0.03).

The inclusion criteria were fever of >38°C and signs and symptoms of acute respiratory infection. Known to the adjudicator were, in addition to clinical findings, CRP, white blood cell counts, X-ray findings, and in some cases, microbiological test results. Based on this information, patients were judged to have either a bacterium or virus as their infectious agent, i.e., they were given a clinical diagnosis of infection. Lower respiratory tract infection was verified by X-ray examination of the lungs. Upper respiratory tract infections were patients with typical symptoms and a negative X ray. Also, patients with typical symptoms of upper respiratory infection, but in whom X-ray examinations of the lungs were not judged to be clinically indicated, were classified as having upper respiratory infections. Exclusion criteria were mycoplasma pneumonia and known chronic viral infection, such as human immunodeficiency virus infection. In addition, children under the age of 18 years and patients who could not give informed consent were excluded from this study. The patients were admitted to the infectious disease department at the University Hospital in Uppsala, Sweden, or to a primary care unit in Uppsala. Blood was drawn before the start of antibiotic treatment. The study was approved by the ethics committee of Uppsala.

In 98 of 168 patients, the cause of infectious agent was verified by objective testing, including positive culture or PCR testing of samples from the respiratory tract and IgG/IgM serology.

### Assay of HNL.

HNL was measured after preactivation for 1 to 5 min of heparinized blood with 5 × 10^−8^ mol/liter tripeptide formyl-methionine-leucine-phenylalanine (fMLP) (BioXtra; Sigma-Aldrich, St. Louis, MO, USA). Ice-cold EDTA was added to stop the release, after which plasma was obtained by centrifugation of the blood. Measurements in plasma were performed by means of a double monoclonal enzyme-linked immunosorbent assay (ELISA) kit provided by Diagnostics Development (Uppsala, Sweden) and run according to the instructions of the provider. The imprecision of the ELISA was <6% coefficient of variation (CV). HNL in whole blood was assayed as described previously (2, 6). Briefly, preactivation of whole blood was performed by incubation of whole blood with 10^−7^ mol/liter fMLP for 20 min at 37°C, after which the blood was applied to the cartridge of a prototype point-of-care (POC) assay on the Meritas platform (Trinity Diagnostics, Uppsala, Sweden).

### Statistics.

Data are expressed as medians and interquartile ranges. Comparisons of groups were performed by the nonparametric Mann-Whitney test for independent groups. In order to estimate the clinical performances of the biomarker assays, receiver operating characteristic (ROC) analyses were performed, and comparisons of the areas under the curve were analyzed by c-statistics. The Youden index (*J*) was calculated by the formula *J* = maximum (sensitivity + specificity − 1) and used to estimate the optimal discriminatory concentration of the biomarker. Based on this index, both the positive likelihood ratio (LR+) and negative likelihood ratio (LR−) were calculated.

For calculations of the statistics, MedCalc Statistical Software version 16.4.3 (MedCalc Software bvba, Ostend, Belgium [https://www.medcalc.org]; 2016) was used.

## References

[1] Yu Z, Jing H, Hongtao P, Furong J, Yuting J, Xu S, Venge P 2016 Distinction between bacterial and viral infections by serum measurement of human neutrophil lipocalin (HNL) and the impact of antibody selection. J Immunol Methods 432:82–86. doi:10.1016/j.jim.2016.02.014. 26899825

[2] Venge P, Douhan-Hakansson L, Garwicz D, Peterson C, Xu S, Pauksen K 2015 Human neutrophil lipocalin as a superior diagnostic means to distinguish between acute bacterial and viral infections. Clin Vaccine Immunol 22:1025–1032. doi:10.1128/CVI.00347-15. 26135974 PMC4550662

[3] Venge P, Hakansson LD, Garwicz D, Peterson C, Xu S, Pauksen K 2015 Human neutrophil lipocalin in fMLP-activated whole blood as a diagnostic means to distinguish between acute bacterial and viral infections. J Immunol Methods 424:85–90. doi:10.1016/j.jim.2015.05.004. 26002155

[4] Xu SY, Pauksen K, Venge P 1995 Serum measurements of human neutrophil lipocalin (HNL) discriminate between acute bacterial and viral infections. Scand J Clin Lab Invest 55:125–131. doi:10.3109/00365519509089604. 7667605

[5] Bjorkqvist M, Kallman J, Fjaertoft G, Xu S, Venge P, Schollin J 2004 Human neutrophil lipocalin: normal levels and use as a marker for invasive infection in the newborn. Acta Paediatr 93:534–539. 15188983

[6] Venge P, Hakansson LD, Garwicz D, Peterson C, Xu S, Pauksen K 2015 Human neutrophil lipocalin in fMLP-activated whole blood as a diagnostic means to distinguish between acute bacterial and viral infections. J Immunol Methods 424:85–90. doi:10.1016/j.jim.2015.05.004. 26002155

[7] Laxminarayan R, Duse A, Wattal C, Zaidi AK, Wertheim HF, Sumpradit N, Vlieghe E, Hara GL, Gould IM, Goossens H, Greko C, So AD, Bigdeli M, Tomson G, Woodhouse W, Ombaka E, Peralta AQ, Qamar FN, Mir F, Kariuki S, Bhutta ZA, Coates A, Bergstrom R, Wright GD, Brown ED, Cars O 2013 Antibiotic resistance–the need for global solutions. Lancet Infect Dis 13:1057–1098. doi:10.1016/S1473-3099(13)70318-9. 24252483

[8] Dupuy AM, Philippart F, Pean Y, Lasocki S, Charles PE, Chalumeau M, Claessens YE, Quenot JP, Guen CG, Ruiz S, Luyt CE, Roche N, Stahl JP, Bedos JP, Pugin J, Gauzit R, Misset B, Brun-Buisson C, Maurice Rapin Institute Biomarkers Group 2013 Role of biomarkers in the management of antibiotic therapy: an expert panel review: I–currently available biomarkers for clinical use in acute infections. Ann Intensive Care 3:22. doi:10.1186/2110-5820-3-22. 23837559 PMC3708786

[9] Hopstaken RM, Muris JW, Knottnerus JA, Kester AD, Rinkens PE, Dinant GJ 2003 Contributions of symptoms, signs, erythrocyte sedimentation rate, and C-reactive protein to a diagnosis of pneumonia in acute lower respiratory tract infection. Br J Gen Pract 53:358–364. 12830562 PMC1314594

[10] Ip M, Rainer TH, Lee N, Chan C, Chau SS, Leung W, Leung MF, Tam TK, Antonio GE, Lui G, Lau TK, Hui DS, Fuchs D, Renneberg R, Chan PK 2007 Value of serum procalcitonin, neopterin, and C-reactive protein in differentiating bacterial from viral etiologies in patients presenting with lower respiratory tract infections. Diagn Microbiol Infect Dis 59:131–136. doi:10.1016/j.diagmicrobio.2007.04.019. 17662560

[11] Toikka P, Irjala K, Juven T, Virkki R, Mertsola J, Leinonen M, Ruuskanen O 2000 Serum procalcitonin, C-reactive protein and interleukin-6 for distinguishing bacterial and viral pneumonia in children. Pediatr Infect Dis J 19:598–602. doi:10.1097/00006454-200007000-00003. 10917215

[12] Le BJ, Hausfater P, Chenevier-Gobeaux C, Blanc FX, Benjoar M, Ficko C, Ray P, Choquet C, Duval X, Claessens YE, ESCAPED Study Group 2015 Diagnostic accuracy of C-reactive protein and procalcitonin in suspected community-acquired pneumonia adults visiting emergency department and having a systematic thoracic CT scan. Crit Care 19:366. doi:10.1186/s13054-015-1083-6. 26472401 PMC4608327

[13] Müller B, Becker KL 2001 Procalcitonin: how a hormone became a marker and mediator of sepsis. Swiss Med Wkly 131:595–602. 11820070 10.4414/smw.2001.09751

[14] Meili M, Muller B, Kulkarni P, Schutz P 2015 Management of patients with respiratory infections in primary care: procalcitonin, C-reactive protein or both? Expert Rev Respir Med 9:587–601. doi:10.1586/17476348.2015.1081063. 26366806

[15] Drozdov D, Dusemund F, Muller B, Albrich WC 2013 Efficacy and safety of procalcitonin-guided antibiotic therapy in lower respiratory tract infections. Antibiotics (Basel) 2:1–10. doi:10.3390/antibiotics2010001. 27029288 PMC4790294

[16] Mårtensson J, Bell M, Xu S, Bottai M, Ravn B, Venge P, Martling CR 2013 Association of plasma neutrophil gelatinase-associated lipocalin (NGAL) with sepsis and acute kidney dysfunction. Biomarkers 18:349–356. doi:10.3109/1354750X.2013.787460. 23627612

[17] Hopstaken RM, Stobberingh EE, Knottnerus JA, Muris JW, Nelemans P, Rinkens PE, Dinant GJ 2005 Clinical items not helpful in differentiating viral from bacterial lower respiratory tract infections in general practice. J Clin Epidemiol 58:175–183. doi:10.1016/j.jclinepi.2004.08.004. 15680752

[18] Xu S, Venge P 2000 Lipocalins as biochemical markers of disease. Biochim Biophys Acta 1482:298–307. doi:10.1016/S0167-4838(00)00163-1. 11058770

[19] Cai L, Rubin J, Han W, Venge P, Xu S 2010 The origin of multiple molecular forms in urine of HNL/NGAL. Clin J Am Soc Nephrol 5:2229–2235. doi:10.2215/CJN.00980110. 20829422 PMC2994084

[20] Xu S, Cai L, Zhao L, Douhan-Hakansson L, Kristjansson G, Pauksen K, Venge P 2010 Tissue localization and the establishment of a sensitive immunoassay of the newly discovered human phospholipase B-precursor (PLB-P). J Immunol Methods 353:71–77. doi:10.1016/j.jim.2010.01.005. 20093120

[21] Mishra J, Dent C, Tarabishi R, Mitsnefes MM, Ma Q, Kelly C, Ruff SM, Zahedi K, Shao M, Bean J, Mori K, Barasch J, Devarajan P 2005 Neutrophil gelatinase-associated lipocalin (NGAL) as a biomarker for acute renal injury after cardiac surgery. Lancet 365:1231–1238. doi:10.1016/S0140-6736(05)74811-X. 15811456

[22] Cai L, Borowiec J, Xu S, Han W, Venge P 2009 Assays of urine levels of HNL/NGAL in patients undergoing cardiac surgery and the impact of antibody configuration on their clinical performances. Clin Chim Acta 403:121–125. doi:10.1016/j.cca.2009.01.030. 19361453

